# Physicochemical properties, pharmacokinetic studies, DFT approach, and antioxidant activity of nitro and chloro indolinone derivatives

**DOI:** 10.3389/fchem.2024.1360719

**Published:** 2024-03-18

**Authors:** Abdul Saboor Pirzada, Haroon Khan, Waqas Alam, Hany W. Darwish, Ahmed A. Elhenawy, Aleksey Kuznetsov, Maria Daglia

**Affiliations:** ^1^ Department of Pharmacy, Abdul Wali Khan University Mardan, Mardan, Pakistan; ^2^ Department of Pharmaceutical Chemistry, College of Pharmacy, King Saud University, Riyadh, Saudi Arabia; ^3^ Chemistry Department, Faculty of Science, Al-Azhar University, Nasr City, Cairo, Egypt; ^4^ Department of Chemistry, Universidad Técnica Federico Santa Maria, Santiago, Chile; ^5^ Department of Pharmacy, University of Napoli Federico II, Naples, Italy; ^6^ International Research Center for Food Nutrition and Safety, Jiangsu University, Zhenjiang, China

**Keywords:** indolinone derivatives, ADME study, density function theory study, antioxidant assays, therapeutic potential

## Abstract

The process of developing of new drugs is greatly hampered by their inadequate physicochemical, pharmacokinetic, and intrinsic characteristics. In this regard, the selected chloro indolinone, (Z)-6-chloro-3-(2-chlorobenzylidene)indolin-2-one (C1), and nitro indolinone, (Z)-6-chloro-3-(2-nitrobenzylidene)indolin-2-one (C2), were subjected to SwissADME and density function theory (DFT) analysis. For compounds C1 and C2, the BOILED-Egg pharmacokinetic model predicted intestinal absorption, blood–brain barrier (BBB) penetration, and p-glycoprotein interaction. According to the physicochemical analysis, C1 has exceptional drug-like characteristics suitable for oral absorption. Despite only being substrates for some of the major CYP 450 isoforms, compounds C1 and C2 were anticipated to have strong plasma protein binding and efficient distribution and block these isoforms. The DFT study using the B3LYP/6-311G(d,p) approach with implicit water effects was performed to assess the structural features, electronic properties, and global reactivity parameters (GRP) of C1 and C2. The DFT results provided further support for other studies, implying that C2 is more water-soluble than C1 and that both compounds can form hydrogen bonds and (weak) dispersion interactions with other molecules, such as solvents and biomolecules. Furthermore, the GRP study suggested that C1 should be more stable and less reactive than C2. A concentration-dependent 2,2-diphenyl-1-picrylhydrazyl (DPPH) and 2,2′-azino-bis(3-ethylbenzothiazoline-6-sulfonic acid (ABTS) radical scavenging activity was shown by both C1 and C2. In brief, this finding has provided a strong foundation to explore further the therapeutic potential of these molecules against a variety of human disorders.

## 1 Introduction

Oxindoles are a class of natural organic heterocyclic compounds found in mammalian tissues and a variety of plant species (Khetmalis et al., 2021). The bark of the tropical climber *Uncaria tomentosa* was used, which led to the isolation of an alkaloid as the first oxindole derivative (Rudrangi et al., 2011). The ethnopharmacology of *U. tomentosa* showed a variety of antiquated treatments for gastric ulcers, cancer, mild physical inflammations, and infections (Laus et al., 1997). Oxindoles have a wide variety of pharmacological activities. Metastatic renal cell carcinoma and gastrointestinal stromal tumors have both been successfully treated with indo-lin-2-one (sunitinib) (Kaur et al., 2016). Indolidan and adibendan, two oxindole derivatives with potent vasodilatory, pro-inotropic, and inodilatory effects, are used to treat congestive heart failure (Rudrangi et al., 2011). Similarly, researchers have established the uses of oxindoles against infection, cancer, gastric ulcer, arthritis, and other mild physical inflammation (Hopkins et al., 2008; Kaur et al., 2016).

According to the IUPAC system of scientific nomenclature, oxindoles are 1,3-dihydro-2H-indole-2-ones with the chemical formula C_8_H_7_NO. Their structure consists of a pyrrole and a benzene ring bonded at the C-2 position by a carbonyl group (Dreifuss et al., 2010). The fully substituted carbon atom of oxindoles is what really gives them their effectiveness (Khetmalis et al., 2021). The pharmacokinetic profile of newly investigated compounds is the key to drug discovery processes. In this regard, SwissADME is a highly used and recommended tool to assess various pharmacokinetic profiles of test compounds (Ahmad et al., 2023). Computational techniques, particularly DFT methods, have been effectively applied to a variety of compounds, from basic organic compounds to multidimensional structures. They aid in understanding the fundamental mechanisms underlying the reactivity characteristics of molecular systems (Othman et al., 2021). DFT methods were used to analyze the monomers and dimers of the substituted oxindoles and predict their electronic, non-linear optical, and reactivity properties (Isik and Sagdinc, 2021). Additionally, experimental and DFT approaches were jointly used to examine the pyrazole derivatives containing the oxindole moiety in order to determine their bioavailability; the study results showed the possibility for these derivatives to act as anti-infection agents (El-Qaliei et al., 2022). It was found that the π-aromatic structure plays a crucial role in the stabilization of the transition states and cycloadducts when using the DFT approach to search for the anti-tumor property of an oxindole moiety functionalized by the C_20_ fullerene. The careful assessment of molecular characteristics is a key strategy in drug development. Such molecular characteristics control a compound’s route inside a living system. Consequently, it is highly beneficial to develop a drug pharmacokinetic profile (Kadhim et al., 2022).

To develop oxindole derivatives having desired biological activities, both natural and synthetic procedures are used. The pharmaceutical industry and academia both have shown greater interest in developing new oxindole compounds having a unique pharmacological profile and strong efficacy (Khetmalis et al., 2021). By reacting 6-chlorooxindole with various aromatic aldehydes in the presence of piperidine, a series of 6-chloro-3-oxindole derivatives 1–25 were produced in high yields by Momin et al. Seven compounds were discovered to be highly effective in inhibiting yeast α-glucosidase, exhibiting various degrees of inhibition. The interactions between the active chemicals and the enzyme were discovered with the use of docking studies. This work has led to the discovery of oxindoles, a previously unreported novel class of α-glucosidase inhibitors (Khan et al., 2014).

The purpose of the current study is to characterize the physicochemical properties, find the pharmacokinetic profiles, and reveal the structural, electronic, and reactivity features of two selected chlorinated oxindole derivatives. Furthermore, their antioxidant activity was investigated. Moreover, a detailed DFT study was performed, and its results were compared with the results obtained by other methods.

## 2 Materials and methods

### 2.1 Compound descriptors

ChemDraw Professional 16.0 was used to create the two-dimensional chemical structures, saved for subsequent use. A structure-based search and a streamlined molecular input line entry mechanism are both supported by the drug-likeness and pharmacokinetics databases (SMILES). By using the SwissADME database, the SMILES notation of each drug was initially obtained. Each molecule’s SMILES notation was used to predict various cheminformatics-related features, establish molecular fingerprints, and assign IUPAC names. International chemical identifier (InChI) software version 1.06 obtained from IUPAC was used to generate InChI and InChI keys (Goodman et al., 2021).

### 2.2 Physicochemical, drug-likeness, and pharmacokinetic characteristics

Physicochemical characteristics and drug-likeness studies and an assessment of the pharmacokinetic properties of compounds can all be performed in virtual laboratories (Ahmad et al., 2023). To evaluate the physicochemical profile, drug-likeness, and pharmacokinetic parameters of oxindole derivatives, the SwissADME database was used (Ahmad et al., 2021). The database was used to predict variables pertaining to pharmacokinetics, medicinal chemistry, and physicochemical features. All these characteristics are taken into account during the drug discovery process, and failure to meet these criteria results in drug rejection. In this regard, several physicochemical attributes were identified and recorded. Moreover, the lipophilicity, BOILED-egg chart, drug-likeness, and medicinal chemistry of C1 and C2 were also determined by the Swiss ADME database.

### 2.3 Computational details

The DFT study was conducted via Gaussian 16 software (Frisch et al., 2016). The hybrid B3LYP functional (Beeke, 1993), which includes Becke’s three-parameter exchange functional along with the Lee, Yang, and Parr correlation functional, in conjunction with the full-electron 6-311G(d,p) basis set (McLean and Chandler, 1980), was employed (this basis set has two sets of polarization functions, on hydrogens and on heavier atoms). All calculations were done by taking into account the implicit effects of H_2_O (dielectric constant ε(H_2_O) = 78.3553). For this, the self-consistent reaction field IEF-PCM method (Tomasi et al., 2005), as implemented in Gaussian 16, was used, with the UFF default model, where the electrostatic scaling factor a was set to 1.0. Analysis of atomic charges was done using the natural bond orbital (NBO) scheme as realized in Gaussian 16N (Reed et al., 1988). Molecular orbitals (MOs) for the optimized molecules were calculated at the B3LYP/6-311G(d,p) level with the implicit water effects. Moreover, time-dependent DFT (TDDFT) calculations (Bauernschmitt and Ahlrichs, 1996) involving the implicit effects of water were done using the TD-B3LYP/6-311G(d,p) approach on the geometry optimized at the B3LYP/6-311G(d,p) level with the implicit effects of water.

Furthermore, we used the energies of the frontier molecular orbitals (FMOs), the highest occupied molecular orbital (HOMO) and the lowest unoccupied molecular orbital (LUMO), along with the HOMO/LUMO energy gap values, to define the global reactivity parameters (Geerlings et al., 2003) of the compounds C1 and C2 (see Eqs 1–6). To compute the ionization potential (*IP*) and electron affinity (*EA*) values, Eqs 1, 2 were used:

$$\text{IP}=‐E_{\text{HOMO}},$$ (1)

$$\text{EA}=‐E_{\text{LUMO}}.$$ (2)

The values of the global hardness *η* and electronegativity X were calculated using Eqs 3, 4:

$$\eta =\frac{\left[IP-EA\right]}{2}=-\frac{\left[E_{LUMO}-E_{HOMO}\right]}{2},$$ (3)

$$X=\frac{\left[IP+EA\right]}{2}=-\frac{\left[E_{\text{LUMO}}+E_{\text{HOMO}}\right]}{2}.$$ (4)

The global electrophilicity values were computed using Eq. 5:

$$\omega =\frac{\mu^{2}}{2\eta },$$ (5)

where $\mu =\frac{E_{HOMO}+E_{LUMO}}{2}$ is the chemical potential.

Finally, the global softness values were calculated using Eq. 6:

$$\sigma =\frac{1}{2\eta }.$$ (6)

The molecular structures, FMOs, and MEP plots were visualized via Avogadro 1.1.1 visualization software (Hanwell et al., 2012; Putz and Mingos, 2013). The electron and hole density distribution (EDD and HDD) concepts were calculated using Multiwfn 3.8 software. EDD and HDD can be explained by MO wavefunction (Φ) and the configuration coefficient (w), which resemble the transition of an electron from an occupied MO (i) to a virtual MO (1), as shown in the equations below:

$$\rho \text{ele}\left(r\right)={\left.{\sum (W}_{i}^{l}\right)}^{2}\;\Phi_{l}\left(r\right)\Phi_{l}\left(r\right)+{\sum \sum W}_{i}^{l}\;{W_{i}^{m}\Phi }_{l}\left(r\right)\Phi_{m}\left(r\right),$$ (7)

$$\rho \text{hole}\left(r\right)={\left.{\sum (W}_{i}^{l}\right)}^{2}\;\Phi_{l}\left(r\right)\Phi_{l}\left(r\right)+{\sum \sum W}_{i}^{l}\;{W_{i}^{l}\Phi }_{l}\left(r\right)\Phi_{m}\left(r\right).$$ (8)

The electron and hole density distribution maps of **C1** and **C2** were calculated using the aforementioned method. The B3LYP/6-311++G(d,p) levels were calculated. The topology analysis was examined through an atom in molecule (AIM) theory. Multiwfn software was used to calculate the topological parameters and electron localization function (Sabud et al., 2023).

### 2.4 *In vitro* antioxidant assay

According to the protocol previously described, an *in vitro* antioxidant assay of the produced compounds was carried out against different enzymes.

#### 2.4.1 DPPH free radical scavenging assay

1,1-diphenyl, 2-picrylhydrazyl (DPPH) free radicals were used to assess the compound’s capacity to scavenge free radicals (Kaleem et al., 2015). Solutions (0.1 mL) with various concentrations (250, 500, and 1000 g/mL) were added to a 0.004% DPPH methanolic solution. By using a UV spectrophotometer, the absorbance at 517 nm was determined after 30 min. Ascorbic acid served as the positive control. The quenching results (%) were recorded as

$$\left[A0‐A1/A0\right]\times 100,$$

where A_0_ stands for the absorbance of the control and A_1_ stands for the absorbance of the chemical sample. The GraphPad Prism program^1^ (GraphPad) was used to create inhibition curves, and median inhibitory dose (IC_50_) values were calculated for each experiment in triplicate.

#### 2.4.2 ABTS free radical scavenging assay

The C1 and C2 antioxidant activity was also verified using 2,2-azino-bis[3-ethylbenzthiazoline]-6-sulfonic acid (ABTS) free radicals. The antioxidant ability to scavenge the ABTS^⋅+^ radical cations, which results in a decrease in absorbance at 734 nm, provides the basis for the action. Solutions of ABTS (7 mM) and K_2_S_2_O_4_ (2.45 mM) were prepared and combined. To obtain a dark-colored solution containing ABTS^⋅+^ radical cations, the resulting mixture was kept at room temperature and in the dark for 12–16 h. To achieve an absorbance value of 0.70 at 734 nm in the assay, phosphate buffer (0.01 M) was added to the ABTS^⋅+^ radical cation solution having pH 7.4.

By combining 300 µL of the compounds with 3.0 mL of the ABTS^⋅+^ solution in a cuvette, the compound’s capacity to scavenge radicals was examined. After mixing the solutions for 1 minute, the decrease in absorbance was monitored spectrophotometrically for 6 minutes (Ahmad et al., 2023). The positive control was ascorbic acid. The assay was performed in triplicate, and the following formula was used to compute the % inhibition:

% scavenging effect = control absorbance–sample absorbance/control absorbance × 100.

The antioxidant action was represented as an inhibition percentage and as an EC_50_ (compound concentration needed for 50% inhibition of ABTS).

## 3 Results

### 3.1 Compound descriptors

The C1 and C2 structures were drawn with ChemDraw Professional 16.0. The IUPAC names of oxindole derivatives are as follows: C1, (Z)-6-chloro-3-(2-chlorobenzylidene)indolin-2-one, whereas for C2, (Z)-6-chloro-3-(2-nitrobenzylidene)indolin-2-one (Table 1).

**TABLE 1:** IUPAC names and structures of C1 and C2.

Compound	IUPAC name	Structure
**C1**	(Z)-6-chloro-3-(2-chlorobenzylidene)indolin-2-one	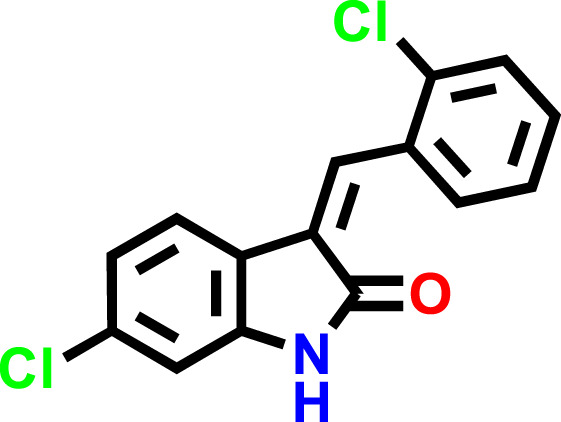
**C2**	(Z)-6-chloro-3-(2-nitrobenzylidene)indolin-2-one	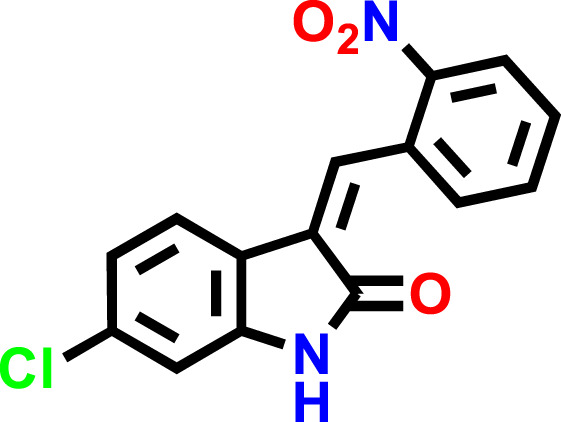

### 3.2 Analysis of physicochemical properties and oral bioavailability radar chart

The physicochemical properties of the synthesized compounds show that these compounds obeyed the Lipinski rule of five (Table 2). This suggests that their molecular composition makes them suitable for oral medications. The oral bioavailability of a pharmacologically suitable molecule is increased when the Lipinski rule of five is followed. C1 and C2 have the respective molecular weights of 290.14 and 300.70 g/mol, respectively, and therefore they are more appropriate to be formulated for oral administration. The number of hydrogen bond donors is 1, while the number of hydrogen bond acceptors (nHA) is 1 for C1 and 3 for C2. C1 has one rotatable bond, while C2 has two. Both C1 and C2 are not formally charged. The ideal charge range is −4∼4 as standardized for a drug candidate. The topological polar surface area (TPSA) for C1 is 29.10 Å^2^, whereas the TPSA for C2 is 74.92 Å^2^. C1 has an electron-donating group with 82.16 M refractive index, whereas C2 has an electron-withdrawing group with an 85.97 M refractive index.

**TABLE 2:** Physicochemical properties of oxindole derivatives C1 and C2 defined with the SwissADME database.

Compound	Molecular formula	MW (g/mol)	nHA	nAHA	F. Csp^3^	nRB	nHBA	nHBD	MR	TPSA (A^2^)
**C1**	C_15_H_9_C_l2_NO	290.14 g/mol	19	12	0.00	1	1	1	82.16	29.10
**C2**	C_15_H_9_C_l_N_2_O_3_	300.70 g/mol	21	12	0.00	2	3	1	85.97	74.92

**FIGURE 1:**
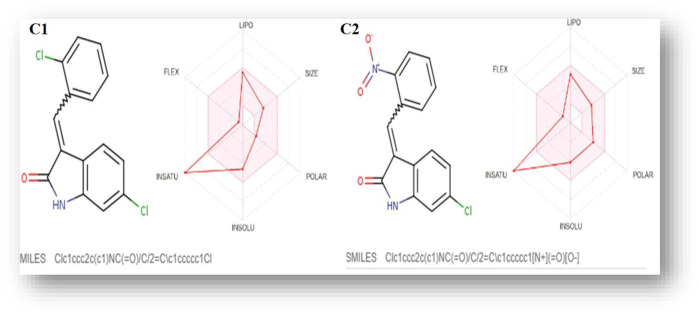
C1 and C2 oral bioavailability radar chart, as predicted using the SwissADME database.

SwissADME was used to predict the oral bioavailability radar map, as shown in Figure 1. The six physicochemical properties of a molecule, namely, flexibility, unsaturation, insolubility, lipophilicity, polarity, and size, are used in this image. Both the compounds’ physicochemical characteristics fall within the range indicated by the pink area, except for the anomalous unsaturation of both compounds. The pink area of the chart represents the appropriate physicochemical environment for orally administered medication.

### 3.3 Solubility and lipophilicity

The lipophilicity and water solubility profiles of C1 and C2 are summarized in Table 3. The aqueous solubility (LogS) of C1 is −4.65, whereas that of C2 is −4.11, both of which are beyond the range of −4∼0.5 log mol/L. With an ideal range of 0–3, logP is the log of the octanol/water partition coefficient. C1 and C2 exhibit logP values of 2.64 and 2.69, respectively, reflecting their partitioning into the lipid compartment. This number matches the LogS shown above. C1 with an electron-donating group displays poor water solubility, whereas C2 with an electron-withdrawing group represents moderate water solubility (Table 3).

**TABLE 3:** Lipophilicity and water solubility of C1 and C2 calculated using the SwissADME database.

Compound	Lipophilicity	Water solubility					
	Consensus log P_o/w_	Log S (ESOL)	Solubility class	Log S (all)	Solubility class	Log S (silicos-it)	Solubility class
**C1**	2.64	−4.65	Moderately soluble	−4.47	Moderately soluble	−6.66	Poorly soluble
**C2**	2.69	−4.11	Moderately soluble	−4.60	Moderately soluble	−5.42	Moderately soluble

### 3.4 Pharmacokinetics parameters and BOILED-Egg chart

The pharmacokinetic parameters of C1 and C2 were analyzed by the SwissADME database, and the results are shown in Table 4. Both C1 and C2 have shown a high gastrointestinal (GI) absorption rate. The bioavailability of C1 and C2 was calculated as 0.55. Both C1 and C2 were reported to cross the blood–brain barrier (BBB). C1 and C2 are not substrates for P-glycoprotein. Cytochrome P450 enzymes are involved in the metabolism of C1 and C2. Both C1 and C2 are found to be inhibitors of CYP 1A2, CYP 2C19, and CYP 2C9.

**TABLE 4:** Pharmacokinetics of oxindole derivatives C1 and C2 calculated with the SwissADME database.

Compounds	GI absorption	Bioavailability score	BBB permeant	P-gp substrate	CYP1A2 inhibitors	CYP2C19 inhibitors	CYP2C9 inhibitors	CYP2D6 inhibitors	CYP3A4 inhibitors
**C1**	High	0.55	Yes	No	Yes	Yes	Yes	No	No
**C2**	High	0.55	Yes	No	Yes	Yes	Yes	No	No

**FIGURE 2:**
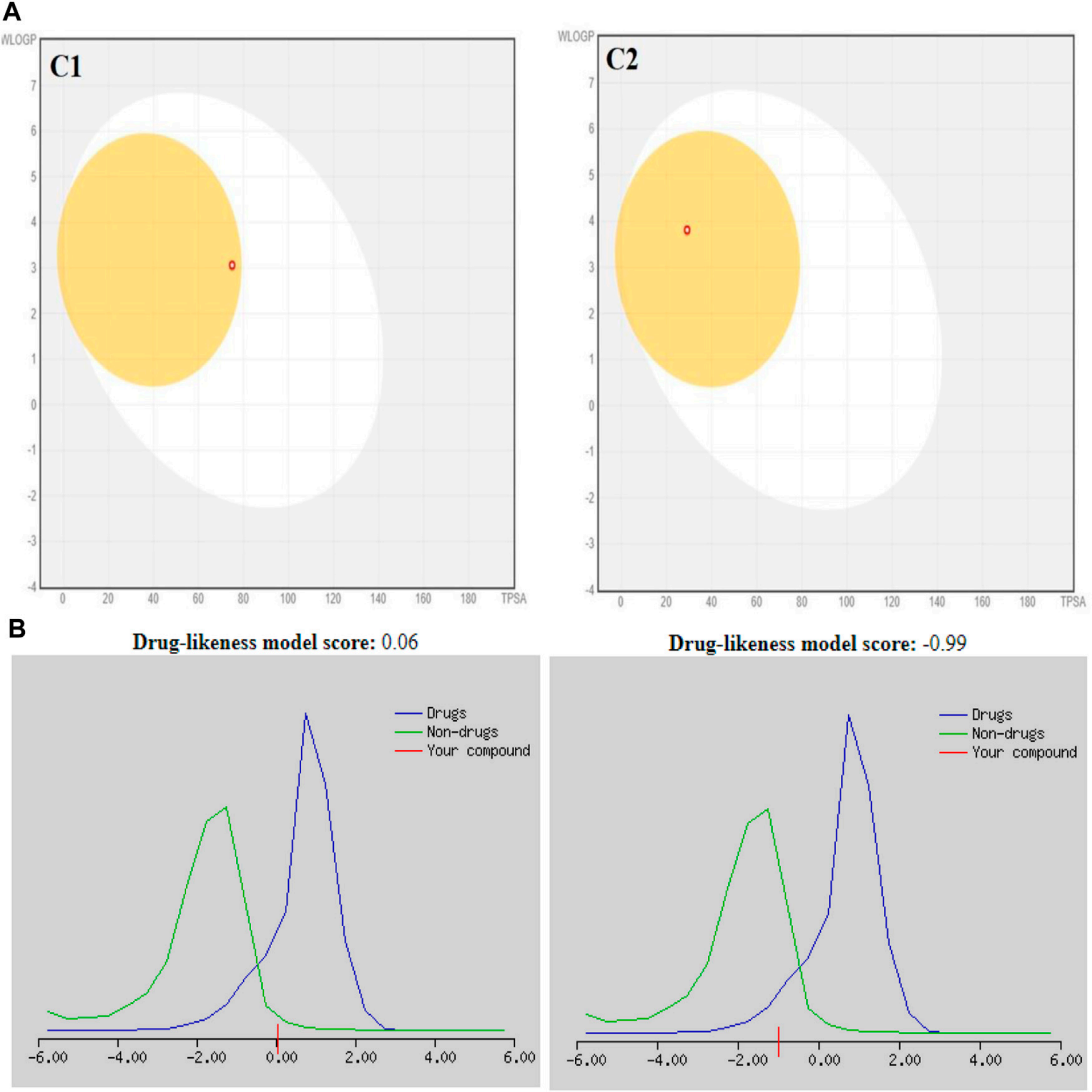
**(A)** Graphical representation for **C1** and **C2** from the SwissADME using the BOILED-Egg method. **(B)** Plotting of the drug-likeness score of **C1** and **C2)** using MolSoft. Non-drug-like behavior (green curve) and drug-like behavior (blue curve).

The SwissADME database provided the BOILED-Egg graphs for both derivatives (Figure 2). Both C1 and C2 were predicted to enter the body through the gastrointestinal tract, cross the BBB, and not act as a P-gp substrate. The gray area of the graph suggests routes other than the oral route, while the egg white part supports human intestinal absorption. Similarly, the egg yellow region suggests entry through the central nervous system (CNS).

### 3.5 Drug likeness and medicinal chemistry

Table 5 shows the SwissADME drug-likeness potential and medicinal chemistry of the tested compounds, C1 and C2. Both C1 and C2 follow all five rules of drug likeness, namely, Lipinski, Ghose, Veber, Egan, and Muegge. There is no violation of drug likeness. The medicinal chemistry profile shows that C1 has two violations (two alerts: michael_acceptor_1 and stilbene) in the Brenk rule, whereas C2 has four violations (four alerts: michael_acceptor_1, nitro_group, oxygen-nitrogen_single_bond, and stilbene). C2 obeys the lead-likeness rules, whereas C1 has one violation, i.e., XLOGP3>3.5. The synthetic accessibility for C1 was 2.48, while that of C2 was 2.62.

**TABLE 5:** Drug-likeness and medicinal chemistry characteristics of C1 and C2 evaluated with the SwissADME database.

Compound	Drug-likeness rules					Medicinal chemistry			
	Lipinski	Ghose	Veber	Egan	Muegge	PAINS	Brenk	Lead likeness	Synthetic accessibility
**C1**	Yes; 0 violation	Yes	Yes	Yes	Yes	0 alert	Two alerts: michael_acceptor_1, stilbene	No; one violation: XLOGP3>3.5	2.48
**C2**	Yes; 0 violation	Yes	Yes	Yes	Yes	0 alert	Four alerts: michael_acceptor_1, nitro_group, oxygen-nitrogen_single_bond, and stilbene	Yes	2.62

Similarly, chloro **C1** and nitro **C2 indolinone** derivatives showed drug-likeness scores equal to 0.06 and −0.99, respectively (Figure 5B).

### 3.6 DFT results

#### 3.6.1 Structural features

**FIGURE 3:**
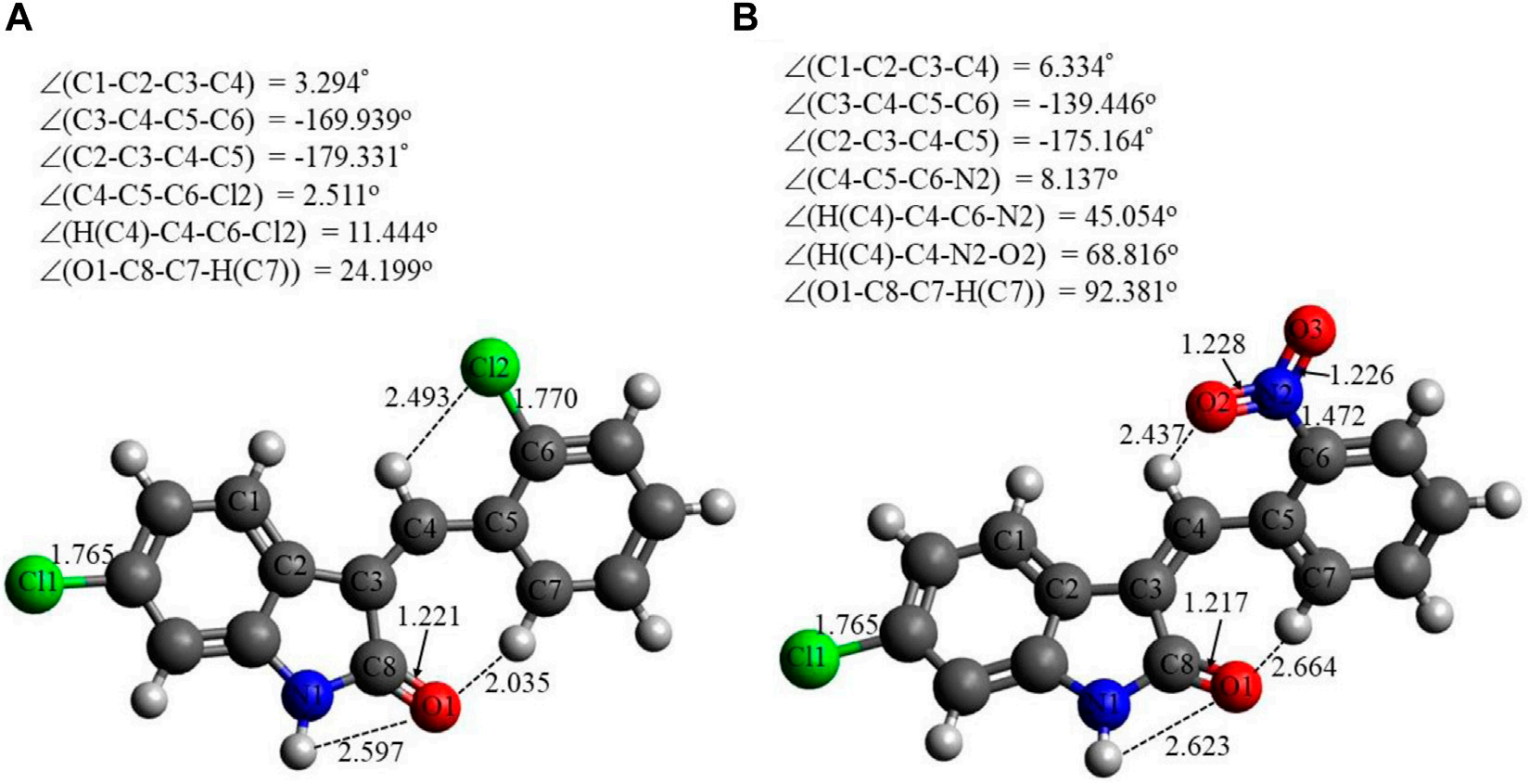
Structures of C1 **(A)** and C2 **(B)** optimized using the B3LYP/6-311G(d,p) approach with the implicit effects of water taken into account. Color coding: dark gray for C, light gray for H, red for O, blue for N, and green for Cl. Selected distances are expressed in Å, and selected dihedral angles are expressed in degrees.

Selected energetic parameters of C1 and C2 calculated at the B3LYP/6-311G(d,p) level with the implicit effects of water are given in Table 6, and their optimized structures along with selected structural parameters (bond distances, interatomic distances, and dihedral angles) are provided in Figure 3. Analysis of the optimized structures shows the following features: (i) C–Cl bond distances in C1 are essentially the same, and C–Cl1 bond distances in C1 and C2 are the same. (ii) C8–O1 bond distances in both compounds are essentially the same. N2–O2 and N2–O3 bond distances in C2 are also essentially the same, suggesting resonance in the nitro group. (iii) In compound C1, the Cl–benzylidene group is quite noticeably tilted relative to the indolin-2-one moiety, as indicated by the dihedral angle ∠(C3–C4–C5–C6) values, which is quite noticeably different from the value of 180^o^. However, in the compound C2, the nitrobenzylidene group is even more strongly tilted relative to the indolin-2-one moiety, as indicated by the values of the dihedral angles ∠(C2–C3–C4–C5) and especially ∠(C3–C4–C5–C6), which is very significantly different from the value of 180^o^. Thus, both compounds are non-flat, and compound C2 is more non-flat than C1. (iv) In C1, there are several relatively short distances between the heavier atoms and the electronegative atoms, such as Cl_2_-H(C4), 2.493 Å; O1-H(N1), 2.597 Å; and O1-H(C7), 2.035 Å, suggesting the potential formation of relatively weak intramolecular noncovalent or hydrogen bonds (see the NBO analysis discussion below). Furthermore, in C2, there are even shorter interatomic distances O2-H(C4), 2.437 Å, but longer interatomic distances O1-H(N1), 2.623 Å, and O1-H(C7), 2.664 Å. These distance values can still suggest the potential formation of relatively weak intramolecular noncovalent or hydrogen bonds (see the NBO analysis discussion below). Moreover, more significant distortion (nonplanarity) of C2 compared to C1 could be related to its higher water solubility (see above).

**TABLE 6:** Selected energetics results for C1 and C2, B3LYP/6-311G(d,p), gas phase//water.

Compound	E_0_, A.U.	E_0_ + ZPE, A.U.	E (HOMO/LUMO), A.U.	E (H/L), eV	TDDFT, eV	Dipole moment, D
C1	−1627.624326//-1627.634325	−1627.423690//-1627.433759	−0.22535/-0.09938//-0.22560/-0.09971	3.43//3.43//	3.02//2.96	1.34//1.55
C2	−1372.552572//-1372.567685	−1372.339807//-1372.355642	−0.23151/-0.11381//-0.22891/-0.10896	3.20//3.27	2.76//2.72	3.11//4.85

#### 3.6.2 Frontier molecular orbital analysis

**FIGURE 4:**
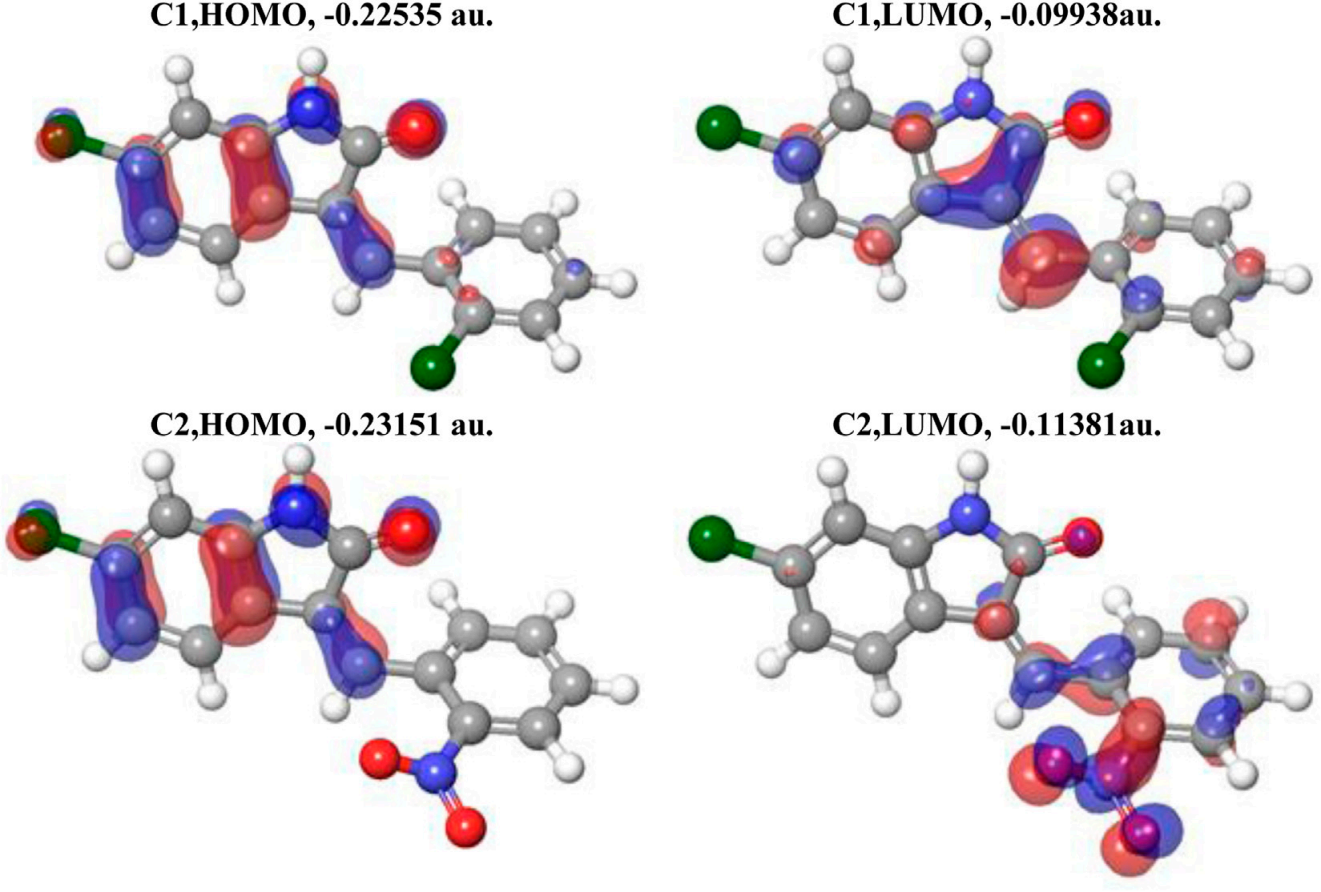
Plots of the HOMO and LUMO of C1 and C2 computed using the B3LYP/6-311G(d,p) approach with the implicit effects of water taken into account. Isosurface values are 0.015 A.U.

In Figure 4, the plots of the FMOs for both compounds are provided, along with the FMO energies in A.U. The FMO plot analysis shows the following features: (i) for both compounds, both the HOMO and LUMO are delocalized over essentially the whole indolin-2-one and can be considered a combination of bonding–antibonding p-orbitals of aromatic moieties and p-orbitals of the chlorine atoms (C1 and C2) and the nitro group (C2). In the case of C2, contributions from both the indolin-2-one and chlorobenzylidene moieties could be qualitatively considered to be of similar magnitude; however, in the case of C1, the HOMO has more significant contributions from the indolin-2-one moiety and the LUMO has more significant contributions from the nitrobenzylidene moiety (with very little contribution from the Cl1 atom, *cf.* Figure 3 for atom numbering). This suggests that for the C1 compound, essentially the whole molecule could act as an electron donor (via its HOMO) or electron acceptor (via its LUMO), but in the case of the C2 compound, the Cl-indolin-2-one moiety would act less as an electron donor (through the C2 HOMO) and the nitrobenzylidene moiety would act more as an electron acceptor (through the C2 LUMO). This would imply some differences in the chemical behavior of both compounds. (ii) The water-calculated C1 HOMO/LUMO gap is somewhat larger than the water-calculated C2 HOMO/LUMO gap, by 0.16 eV, and the corresponding TDDFT gaps are different by 0.24 eV (see Table 3). This would imply higher stability and somewhat lower reactivity of compound C1 compared to compound C2 (see discussion of Global Reactivity Parameters below), which in turn could be related to the relative potential toxicity of these compounds as drug candidates.

#### 3.6.3 NBO charge analysis

**FIGURE 5:**
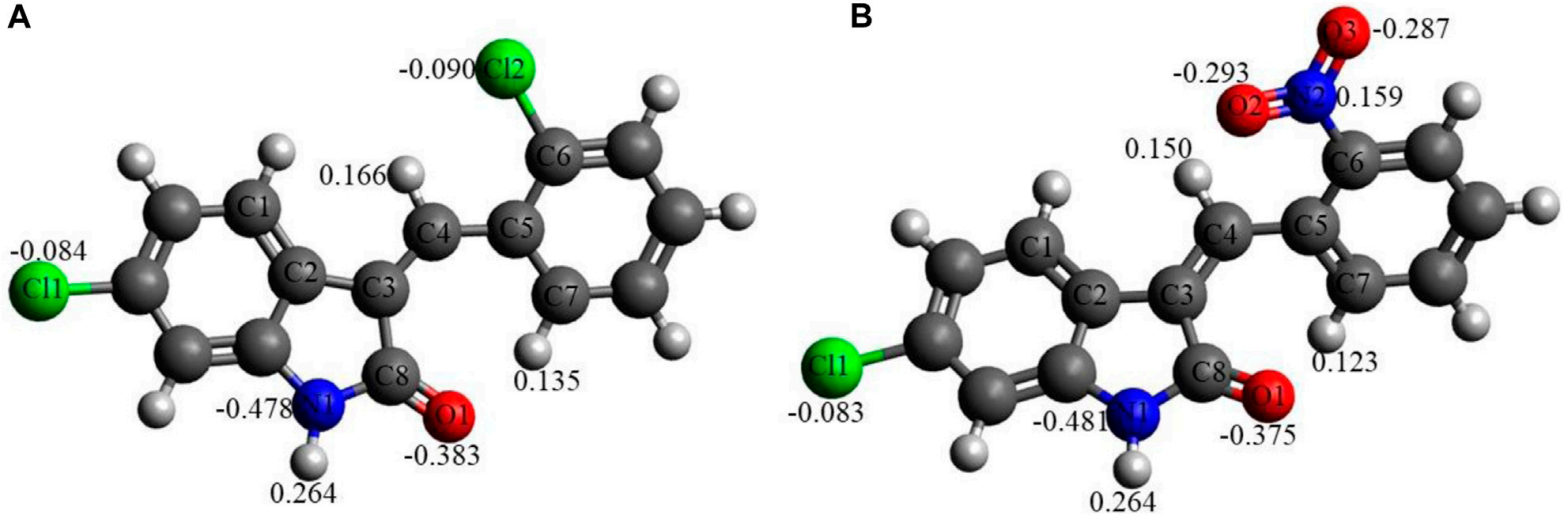
NBO charges, e, on selected atoms for C1 **(A)** and C2 **(B)** obtained using the B3LYP/6-311G(d,p) approach with the implicit effects of water taken into account.

Figure 5 shows the NBO charges on the C1 and C2 selected atoms. Analysis of these charges reveals the following: (i) the Cl atoms in C1 and in C2 have quite similar negative charges, −0.083 to −0.090e. (ii) The O1 atoms in both compounds bear quite significant negative charges, −0.375 to −0.383e. The O atoms of the nitro group in C2 bear significant negative charges as well, −0.287 to −0.293e, with the N atom of the nitro group bearing a noticeable positive charge, 0.159e. (iii) The N1 atoms of both compounds have significant negative charges, −0.478 to −0.481e. (iv) Finally, the selected hydrogens of both compounds, H(N1), H(C4), and H(C7), bear noticeable positive charges, 0.123–0.264e.

Thus, these noticeable charges on the selected atoms suggest the formation of the intramolecular O1-H(N1) hydrogen bond in both compounds and (weak) intramolecular noncovalent interactions O1-H(C7) in both compounds, Cl2-H(4) in C1 and O2-H(C4) in C2 (see above the discussion of structural features). Furthermore, these charges imply intermolecular hydrogen bonding formation and dispersion interactions with solvent molecules for both compounds, in agreement with their solubility analysis (see Section 3.3). The higher water solubility of compound C2 could be explained by the presence of the nitro group with high negative charges on its oxygen (see also consideration in the Structural features subsection). Moreover, these charges on the selected atoms of both compounds would suggest the formation of intermolecular hydrogen bonding (in line with physicochemical analysis results) and dispersion interactions with biomolecules, thus supporting their suitability as drug candidates. Moreover, the noticeably higher dipole moment of the C2 molecule (see Table 6) supports the higher polarity and water solubility of compound 2 as well.

#### 3.6.4 Global reactivity parameter analysis

The global reactivity parameters (GRPs) were computed using the FMO energies (Table 6) using Eqs 1–6 (computational details), and their values in *eV* are given in Table 7.

**TABLE 7:** GRPs for C1 and C2 compounds (*eV*) obtained using the B3LYP/6-311G(d,p) approach with the implicit effects of water.

Ligands	*E (HOMO)*	*E (LUMO)*	*IP*	*EA*	*Gap*	*X*	*η*	*μ*	*σ*	*ω*
**C1**	−6.14	−2.71	6.14	2.71	3.43	4.425	1.715	−4.425	0.292	5.709
**C2**	−6.23	−2.96	6.23	2.96	3.27	4.595	1.635	−4.595	0.305	6.457

The analysis of the GRP parameters shown in Table 7 reveals the following features: (i) both compounds are characterized by relatively high IP and EA values of 6.14 and 6.23 eV and 2.71 and 2.96 eV, respectively. C2 has both IP and EA values higher than those of C1. Thus, C1 should be a somewhat better electron donor than C2, whereas C2 should be a somewhat better electron acceptor, which is in line with the presence of quite an electronegative nitro group in C2. (ii) The global hardness (*η*) value for C1, 1.715 eV, is higher than that for C2, 1.635 eV, which implies less polarizability, lower reactivity, and higher stability of C1 than of C2, which is in line with the higher HOMO/LUMO gap value for C1 compared to C2, 3.43 eV vs 3.27 eV. However, the chemical potential (*μ*) values for these compounds, −4.425 eV and −4.595 eV for C1 and C2, respectively, show the opposite trend, implying that C2 should be more stable. Furthermore, the global softness (*σ*) value for C1, 0.292 eV, is smaller than that for C2, 0.305 eV, implying lower reactivity of C1 than of C2. (iii) The global electronegativity (*X*) for C1, 4.425 eV, is lower than that for C2, 4.595 eV, and the electrophilicity (*ω*) for C1, 5.709 eV, is noticeably lower than that for C2, 6.457 eV, thus implying lower reactivity of C1 than C2. These values are in agreement with the IP and EA values of both compounds.

Thus, the GRP analysis suggests that C1 is a somewhat better electron donor than C2, whereas C2 is a somewhat better electron acceptor, and, in general, less polarizability, lower reactivity, and higher stability for C1 than C2.

#### 3.6.5 MEP surface analysis

**FIGURE 6:**
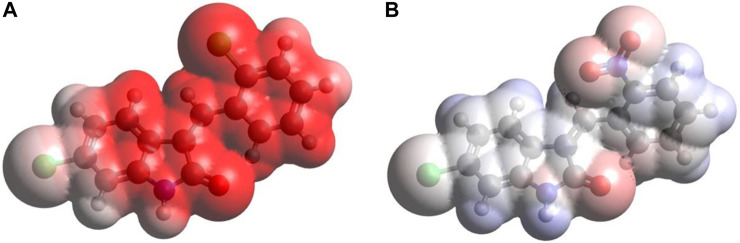
MEP plots for the compound C1 **(A)** and C2 **(B)** computed using the B3LYP/6-311G(d,p) approach with the implicit effects of water taken into account.

Figure 6 provides the MEP plots for both compounds. The MEP plot analysis shows the following features: (i) for C1, most of the molecule accumulates negative electrostatic potential (shown by red color), with somewhat lower negative potential accumulation on the Cl1 atom. (ii) For C2, the situation is drastically different: there are some noticeable accumulations of negative MEP (red color) on the nitro group, O1 atom, and next to the N1 atom, with noticeable accumulations of positive MEP (blue color) on the hydrogens of benzene rings and on the H(N1) atom.

Thus, the MEP analysis implies that both compounds can form intermolecular hydrogen bonding (in line with physicochemical analysis results) and dispersion interactions with solvent molecules and biomolecules. However, for C1, interactions with polar solvent molecules, such as water, would be restricted only by interactions with positively charged hydrogens of water molecules, which might suggest lower water solubility of C1.

#### 3.6.6 Analysis of electron excitation using electron and hole density distributions

**FIGURE 7:**
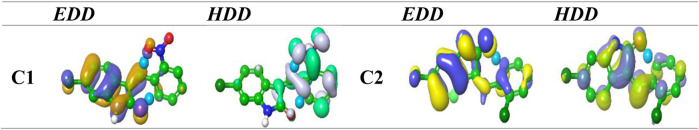
EDD and HDD profiles for the excited state for compounds nitro and chloro indolinone derivatives.

The concept of multi-molecular orbital excitation is based on the distribution of electron and hole density in the receptors and their corresponding receptor anions. This concept reveals the characteristics of the excited state for the **indolinone** (**NO**_**2**_ **and Cl**) molecules. The electron and hole density distribution can also help us understand the electronic structure and properties of these molecules, as well as the mechanisms of molecular recognition and binding in receptor–ligand interactions. The excited state can be modeled by the excitation of an electron from an occupied to a virtual MO. By comparing the ground-state MO with the photo-excited states, we can see the differences in the electron density distribution maps (Figure 7). These maps show the spatial distribution of the electrons and holes in the molecule and provide information about the excited state energy level, the location and nature of excited electrons, and the degree of electron delocalization. The electron and hole density distributions can be calculated by using the MO wave function (Φ) and the configuration coefficient (w), which describe the transition of an electron from an occupied MO (i) to a virtual MO (j), as shown in the equation below:

ρele(r) = Σiw*iΦ*i(r)^ 2Φ_j(r)^ 2 ρhole(r) = Σiw_iΦ.

#### 3.6.7 Topology analysis

**FIGURE 8:**
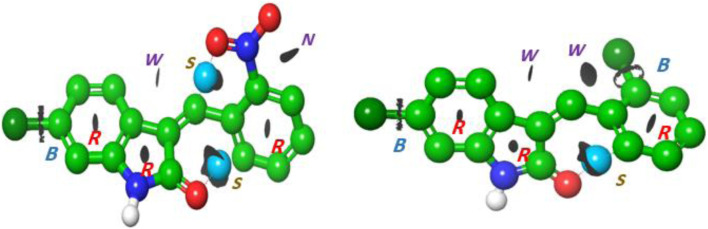
Interaction region indicator surfaces of **C1** and **C2** based on QTAIM analysis, B(BCP), R(RCP); blue dot represents (S) strong interaction; N, noncovalent interaction; and isosurface represents (w) weak interaction.

To investigate the intramolecular interactions within these targeted derivatives, topological analyses of the atoms in each compound were implemented (Figure 8). Bond critical path (BCP), which manifests as a blue isosurface in the atoms in molecule (AIM) assessment, assures the occurrence of bonds between the atoms. The executed BCP for indolinone (NO_2_ and Cl) is illustrated in Figure 2. The BCP associated with C=C•••Cl in (2) and C=C•••C-NH and C=C•••Cl in (1) corroborates the incidence of intramolecular interaction, which stabilizes the molecular structure. The strong interaction, represented by the blue dots, formed between the carbonyl of indoline and C3 of the benzene ring in both compounds, and other strong C=C•••O-N=O interactions formed in the nitro compound. The ring critical path (RCP) formed in three rings that appeared in the center of the surface of these rings is illustrated in Figure 2. The total electron density $\rho \left(r\right)$ and Laplacian ${⊲}^{2}\rho \left(r\right)$ values characterize bond constitution. The total electron density is quantified by the equation

$$\frac{1}{4}{⊲}^{2}\rho \left(r\right)=G\left(r\right)+Y\left\{r\right\},$$

where G(r) and V(r) are Lagrangian kinetic energy and potential energy densities at critical points, respectively. Figure 8 proposes the presence of weak hydrogen bonding as a blue dot between the O of the indolinone ring and nitrophenyl rings. The isosurfaces appear between C=C•••C_6_H_4_ for both compounds, which is indicative of non-covalent hydrogen bond dynamics.

### 3.7 DPPH activity

A concentration-dependent free radical scavenging activity was demonstrated by C1 and C2 (Table 8). At a concentration of 1000 g/mL, ascorbic acid exhibited the maximum scavenging activity of 83.65%, preceded by C2 and C1, at 76% and 72.56%, respectively. In Table 8, several concentrations (1000, 500, 250, 125, and 62.5 g/mL) of C1, C2, and ascorbic acid demonstrated DPPH free radical scavenging in the study. The IC_50_ (µg/mL) values of C1 and C2 *versus* ascorbic acid were determined and are given in Table 8. C1 and C2 exhibited IC_50_ values of 69.375 and 49.033 μg/mL, respectively, compared to ascorbic acid (28.554 μg/mL).

**TABLE 8:** Summary of (%) percent inhibition and IC_50_ of C1 and C2 for DPPH activity.

Concentration (µg/mL)	(%) Percent inhibition	IC_50_ (µM)	(%) Percent inhibition	IC_50_ (µM)	(%) Percent inhibition	IC_50_ (µM)
	Ascorbic acid		C1		C2	
**1000**	83.65 ± 0.67	162.12	72.56 ± 0.58^***^	239.109	76.0 ± 0.77^***^	163.1
**500**	76.67 ± 0.58		70.34 ± 0.78^***^		67.0 ± 0.89^***^	
**250**	69.54 ± 0.33		67.55 ± 0.67^***^		58.0 ± 0.67^***^	
**125**	65.59 ± 0.77		55.43 ± 0.89^**^		51.0 ± 0.87^**^	
**62.5**	58.89 ± 0.78		46.76 ± 0.82^**^		43.0 ± 0.54^**^	

### 3.8 ABTS activity

The *in vitro* ABTS scavenging activity of C1 and C2 is shown in Table 9. A concentration-dependent ABTS cation radical scavenging activity was demonstrated by C1 and C2. C1 demonstrated 77.93% activity at 1000 g/mL, whereas C2 exhibited the maximum scavenging activity at 79.56%. The highest percent inhibition value of gallic acid was found to be 82% at 1000 μg/mL. The *in vitro* IC_50_ values of C1, C2, and gallic acid were also determined and are given in Table 9. The IC_50_ values of C1 (63.089 μg/mL) and C2 (79.337 μg/mL) were comparable to that of the standard drug, gallic acid (58.096 μg/mL).

**TABLE 9:** Summary of (%) percent inhibition and IC_50_ of C1 and C2 for ABTS activity.

Concentration (µg/mL)	(%) Percent inhibition	IC_50_ (µM)	(%) Percent inhibition	IC_50_ (µM)	(%) Percent inhibition	IC_50_ (µM)
	Gallic acid		C1		C2	
**1000**	82.0 ± 0.45	341.50	77.93 ± 0.45^***^	217.44	79.56 ± 0.89^***^	263.84
**500**	77.0 ± 0.67		71.89 ± 0.89^***^		71.45 ± 0.78^***^	
**250**	70.0 ± 0.81		67.98 ± 0.66^***^		67.67 ± 0.67^***^	
**125**	65.0 ± 0.37		60.56 ± 0.61^***^		56.45 ± 0.72^***^	
**62.5**	50.0 ± 0.56		46.92 ± 0.82^***^		45.43 ± 0.56^**^	

## 4 Discussion

In the drug discovery process, every drug must possess a number of physicochemical characteristics that control how it behaves in a biological system before it can be used as a medication. Ninety percent of oral medicines that have progressed to clinical trial phase II are governed by the Lipinski rule of five (RO5), a ubiquitous rule. Four straightforward physicochemical parameters, namely, molecular weight (≤500), number of H-bond acceptors (≤10), number of H-bond donors (≤5), and log P (≤5), define this rule. Both studied compounds are suitable candidates for the development of oral drugs since they meet the RO5 requirements. Both derivatives have sufficient intestinal permeability and good aqueous solubility to attain oral bioavailability. The molecular weights, acceptors/donors of hydrogen bonds, and logP are all within the Lipinski rule’s stringent bounds (Bitew et al., 2021). The majority of oral medications have few rotatable bonds, H-bond donors, and H-bond acceptors. The number of rotatable bond counts is employed as a “drug filter.” Rat oral bioavailability is correlated with greater than 10 rotatable bonds (Daina and Zoete, 2016). Although this filter lacks a molecular basis, it can have an impact on *in vitro* screening. The ligand affinity diminishes for each of the two rotatable bonds by an average of 0.5 k calB (Daina and Zoete, 2016). The ideal limit for the TPSA is 0–140 A^2^, and C1 and C2 have the TPSA within the limits (29.10 Å^2^ and 74.92 Å^2^, respectively). The TPSA up to 60 A^2^ favors >90% absorption, while TPSA ≥ 140 A^2^ has less than 10% of the proportion absorbed (Clark, 1999). The molar refractivity (MR) of both compounds is within the permitted range. The Ghose rule describes molar refractivity and how it affects oral bioavailability. Yet Lipinski’s guideline specified the range of 40–130 for drug likeness (Ansari et al., 2023). The fraction of sp^3^ hybridized carbon atoms, or Fsp^3^, measures the degree to which molecules are saturated with carbon and describes the intricacy of their spatial structures. It is the proportion of sp^3^ hybridized carbons to all carbons (Lovering et al., 2009). In order to predict solubility, Yan and Gesteiger (2003) used Fsp^3^ to assess the degree of molecular aliphaticity. Both derivatives, however, are lacking in sp^3^ hybridized carbons, whose fractions are under the ideal value of ≥0.42 (Kombo et al., 2013). SwissADME was used to predict the oral bioavailability radar map. The six physicochemical properties, namely, molecular flexibility, unsaturation, insolubility, lipophilicity, polarity, and size, are used in this image. Both derivative physicochemical characteristics fall within the range indicated by the pink area, with the exception of the anomalous unsaturation of both compounds. The pink area of the chart shows the appropriate physicochemical environment for oral medication bioavailability. Better intestinal absorption was predicted by the SwissADME, which was also supported by the BOILED-Egg plot. P-gp plays a key role in the absorption and elimination of drugs. Both C1 and C2 are predicted to be free from clinically relevant drug interactions and not to be P-gp substrates because P-gp inducers and/or inhibitors may alter the absorption and excretion of medicines that act as P-gp substrates. This quickly exposes them to medication interactions (Yamazaki et al., 2019). It is predicted that C1 and C2 will both inhibit CYP1A2, CYP2C19, and CYP2C9. Both compounds, however, are solely substrates for one or two CYP 450 isoforms. The fact that C1 and C2 can change the plasma level of a drug metabolized by these CYP 450 isoforms confirms the fact that their blood level may be little or unaffected by other CYP 450 inducers. Pharmacokinetic drug interactions can result from the activation or inhibition of a metabolic enzyme that affects the blood level of a drug that is a substrate for the target enzyme (Telbisz ÁA et al., 2021). The PAINS rules did not detect any alerts. By generating false-positive findings during high-throughput screening, pan-assay interference compounds display a promiscuous propensity. It is unclear how such promiscuous activity occurs. Nevertheless, compounds with PAINS alerts show protein reactivity and noncovalent connections (Bolz et al., 2021). Both compounds showed Brenk alerts, while only C1 exhibited one alert for lead likeness. According to the drug-likeness criterion, we observed that the **C1** indolinone with two chloro substituents had a drug-likeness score of 0.06, suggesting that it is a potential drug candidate, whereas the **C2** indolinone with a nitro substituent had a drug-likeness score of −0.99, indicating that it is not a viable drug candidate. The drug-likeness model score analysis is based on the positive and negative values of the target **C1** and **C2 indolinones**. Positive values characterize an agent as a drug. However, the negative value of the drug-likeness score reflects an agent as a non-drug (Gupta et al., 2019).

The DFT studies explored that (i) the optimized structural parameters for both compounds suggest the potential formation of relatively weak intramolecular noncovalent or hydrogen bonds. The more significant distortion (nonplanarity) of C2 compared to C1 could be related to its higher water solubility. (ii) The FMOs of both compounds imply higher stability and somewhat lower reactivity of compound C1 compared to compound C2, which in turn could be related to the relative potential toxicity of these compounds as drug candidates. (iii) Noticeable charges on the selected atoms of both compounds suggest the formation of the intramolecular O1-H(N1) hydrogen bond in both compounds and (weak) intramolecular noncovalent interactions O1-H(C7) in both compounds, Cl2-H(4) in C1 and O2-H(C4) in C2. Furthermore, these charges imply intermolecular hydrogen bonding formation and dispersion interactions with solvent molecules for both compounds, in agreement with their solubility analysis. The higher water solubility of compound C2 could be explained by the presence of a nitro group with high negative charges on its oxygens. Moreover, these charges on the selected atoms of both compounds would suggest the formation of intermolecular hydrogen bonding (in line with physicochemical analysis results) and dispersion interactions with biomolecules, thus supporting their suitability as drug candidates. Furthermore, the noticeably higher dipole moment of the C2 molecule supports the higher water solubility of compound 2. (iv) The GRPs of both compounds suggest that C1 should be a somewhat better electron donor than C2, whereas C2 should be a somewhat better electron acceptor, and, in general, less polarizability, lower reactivity, and higher stability for C1 than C2 as shown. (v) Finally, the MEP analysis implies that both compounds can form intermolecular hydrogen bonding (in line with physicochemical analysis results) and dispersion interactions with solvent molecules and biomolecules. However, for C1, interactions with polar solvent molecules, such as water, would be restricted only by interactions with positively charged hydrogens in water molecules, which might suggest lower water solubility of C1.

The EDD maps in Figure 7 show a thicker surface on the complete indolinone fragment for **C1** and **C2**, indicating a higher electron density in those regions. The HDD map displays higher hole density which is represented by a denser surface on the full nitrophenyl fragment. A simple and intuitive way to assess how much charge is transferred between the electron and hole regions is to calculate their centroids (C− and C+). The investigation HDD map clearly indicates that the indolinone core has the highest electron density for **C1** and **C2,** which is transferred to the nitrophenyl part, as depicted in the EDD map.

The structures of **C1** and **C2** molecules are influenced by several types of interactions, including covalent, non-covalent, and electrostatic interactions. Reduced density gradient (RDG) analysis can reveal noncovalent interactions (NCIs) and visualize them, employing a fluctuation of gradient isosurface proportionate to the interaction intensity. The sign(λ_2_)ρ values designate the bonding variety, where great negative quantities signify hydrogen bonding (blue dot), great positive quantities signify repulsive interactions (blue isosurface), and quantities near 0 signify weak van der Waals interactions (green isosurface) (Otero-De-La-Roza et al., 2012).

In Figure 8, the blue dot color in molecules revealed the presence of H-interaction at C=O•••O. Simultaneously, the green isosurface indicated the presence of a repulsive interaction between pyrrolidine and phenyl rings for C1 and C2. The green color depicted the presence of weak van der Waals interactions between the Cl atom and C=C for the C2 compound, while in C1, noncovalent interactions formed between the nitro group and phenyl ring. From these results, it is concluded that the manifestation of noncovalent interactions in the investigated compounds enhanced their stability in the biological media.

Direct H-atom transfer and sequential proton loss-electron transfer are the two separate methods used by the stable free radical known as DPPH. By giving hydrogen atoms or electrons, antioxidants squelch DPPH free radicals. The DPPH molecule is being attacked by free radicals, which transforms them into a colorless byproduct. DPPH can receive an electron or a radical without hydrogen since it has an odd number of electrons. Because of proton transfer from the antioxidant molecule, the presence of an antioxidant pairs this odd electron, which lowers the amount of DPPH that is absorbed. The same method is used to measure the amount of MnO_2_ that causes the production of the ABTS^⋅+^ radical cation. The absorbance at 734 nm is used to determine its green color. It is converted to ABTS by an antioxidant molecule’s electron donation radical scavenging effect, preventing the development of the green ABTS radical, which causes the ABTS solution to become discolored (Rudrapal et al., 2022).

## 5 Conclusion

In brief, we analyzed the physicochemical characteristics and pharmacokinetics, performed DFT investigations, and investigated antioxidant profiles of two chlorinated oxindole derivatives C1 and C2. Both compounds exhibited conformity with chemical characteristics indicative of drugs, such as Lipinski RO5, PAINS, Brenk, and the lead-likeness rule. The compounds were established to be suitable for oral absorption, have strong plasma protein binding, and are inhibitors of some important CYP 450 isoforms. Both the compounds were found to possess significant antioxidant activity. The DFT study results provided further support for other studies, implying that C2 is more soluble than C1 and that both compounds can form hydrogen bonding and (weak) dispersion interactions with other molecules, such as solvents and biomolecules. Furthermore, the GRP study suggested that C1 should be more stable and less reactive than C2.

This study’s potential future implications and directions are that the ADME profile of antioxidants is directly proportional; however, a chemical antioxidant assay may not always reflect *in vivo* activities. As a result, we search for potential preliminary pharmacokinetic data that suggest C1 and C2 for further research. The admission of a drug candidate to a drug pool is determined by pharmacokinetics. In this case, the biological pre-screening stage is crucial for computational pharmacokinetics. To further confirm the pharmacological effects of the tested compounds, further *in vitro*, *in vivo*, and detailed clinical investigations are needed.

## Data Availability

The original contributions presented in the study are included in the article/Supplementary material; further inquiries can be directed to the corresponding author.

## Footnotes

1 Swift, Mary L. “GraphPad prism, data analysis, and scientific graphing.” *Journal of chemical information and computer sciences* 37, no. 2 (1997): 411-412.

## Abbreviations

ABTS, 2,2′-azino-bis(3-ethylbenzothiazoline-6-sulfonic acid); BBB, blood–brain barrier; CYP 450, cytochrome p450; DFT, density functional theory; DPPH, 2,2-diphenyl-1-picrylhydrazyl; FMOs, frontier molecular orbitals; IUPAC, International Union of Pure and Applied Chemistry; Log S, aqueous solubility; Log P, octanol–water partition coefficient; MR, molecular refractivity; MW, molecular weight; nRB, number of rotatable bonds; nHBA, number of hydrogen bond acceptors; nHBD, number of hydrogen bond donors; TPSA, topological polar surface area.
